# The role of ecological variation in driving divergence of sexual and non-sexual traits in the red-backed fairy-wren (*Malurus melanocephalus*)

**DOI:** 10.1186/1471-2148-13-75

**Published:** 2013-03-28

**Authors:** Daniel T Baldassarre, Henri A Thomassen, Jordan Karubian, Michael S Webster

**Affiliations:** 1 Department of Neurobiology and Behavior, Cornell University, Ithaca, NY, 14850, USA; 2 Macaulay Library, Cornell Lab of Ornithology, Ithaca, NY, 14853, USA; 3 Institute of Evolution and Ecology, University of Tübingen, Tübingen, D-72076, Germany; 4 Department of Ecology and Evolutionary Biology, Tulane University, New Orleans, LA, 70118, USA

**Keywords:** Generalized dissimilarity modeling, Ecological selection, Sexual selection, Fisher process, Isolation by distance, Speciation, *Malurus*

## Abstract

**Background:**

Many species exhibit geographic variation in sexual signals, and divergence in these traits may lead to speciation. Sexual signals may diverge due to differences in ecology if the environment constrains signal production or transmission. Alternatively, sexual signals may diverge stochastically through sexual selection or genetic drift, with little environmental influence. To distinguish between these alternatives we quantified variation in two putative sexual signals – tail length and plumage color – and a suite of non-sexual morphometric traits across the geographic range of the red-backed fairy-wren (*Malurus melanocephalus*). We then tested for associations between these traits and a number of environmental variables using generalized dissimilarity models.

**Results:**

Variation in morphometric traits was explained well by environmental variation, irrespective of geographic distance between sites. Among putative signals, variation in plumage color was best explained by geographic distance, whereas tail length was best explained by environmental variation. Divergence in male plumage color was not coincident with the boundary between genetic lineages, but was greatest across a contact zone located 300 km east of the genetic boundary.

**Conclusions:**

Morphometric traits describing size and shape have likely been subject to ecological selection and thus appear to track local environmental variation regardless of subspecies identity. Ecological selection appears to have also influenced the evolution of tail length as a signal, but has played a limited role in shaping geographic variation in plumage color, consistent with stochastic divergence in concert with Fisherian selection on this trait. The lack of coincidence between the genetic boundary and the contact zone between plumage types suggests that the sexual plumage signal of one subspecies has introgressed into the genetic background of the other. Thus, this study provides insight into the various ways in which signal evolution may occur within a species, and the geographic patterns of signal variation that can arise, especially following secondary contact.

## Background

Divergence in sexual signals is important to the generation of biodiversity because it may lead directly to assortative mating and reproductive isolation between taxa [1]. Indeed, because sexual signals affect mate recognition and thus pre-mating isolation, their divergence may lead to reproductive isolation more rapidly than the accumulation of post-zygotic barriers [1,2] However, the relative importance of different evolutionary forces in causing variation and divergence of sexual signals remains unclear [3].

Non-sexual traits that are of direct ecological importance, such as those affecting foraging and thermoregulation, are likely to diverge via ecological selection and therefore should track underlying environmental variation [4]. For example, variation in bill length is correlated with environmental differences between the Andean lowlands and highlands in the speckled hummingbird (*Adelomyia melanogenys*), most likely due to different selection regimes imposed by variable flower morphologies [5]. Similarly, sexual signals may be constrained by ecology and environmental variation if, for example, diet affects the expression of the trait [6], or if ecological factors constrain the transmission of signals [7]. In these situations, ecological selection helps shape the sexual signal, and geographic variation in the signal should also track environmental variation.

Alternatively, sexual signals may diverge via stochastic processes independent of environmental variation [8,9]. In these models, random drift in the responses of receivers to variation in sexual signals can cause rapid and stochastic change in the signal, leading to phenotypic variation across populations that is not correlated with ecological factors. For example, Prum [10] found that the explosive radiation of sexual signals in Neotropical manakins (Family: Pipridae) is consistent with a stochastic divergence followed by Fisherian sexual selection with little direct or indirect influence of ecology. There are thus multiple phenotypic axes along which taxa may diverge during speciation, with varying opportunities for environmental influence. The relative importance of ecological selection compared to Fisherian sexual selection, especially during early divergence of sexual signals, is the subject of recent debate [11].

**Figure 1:**
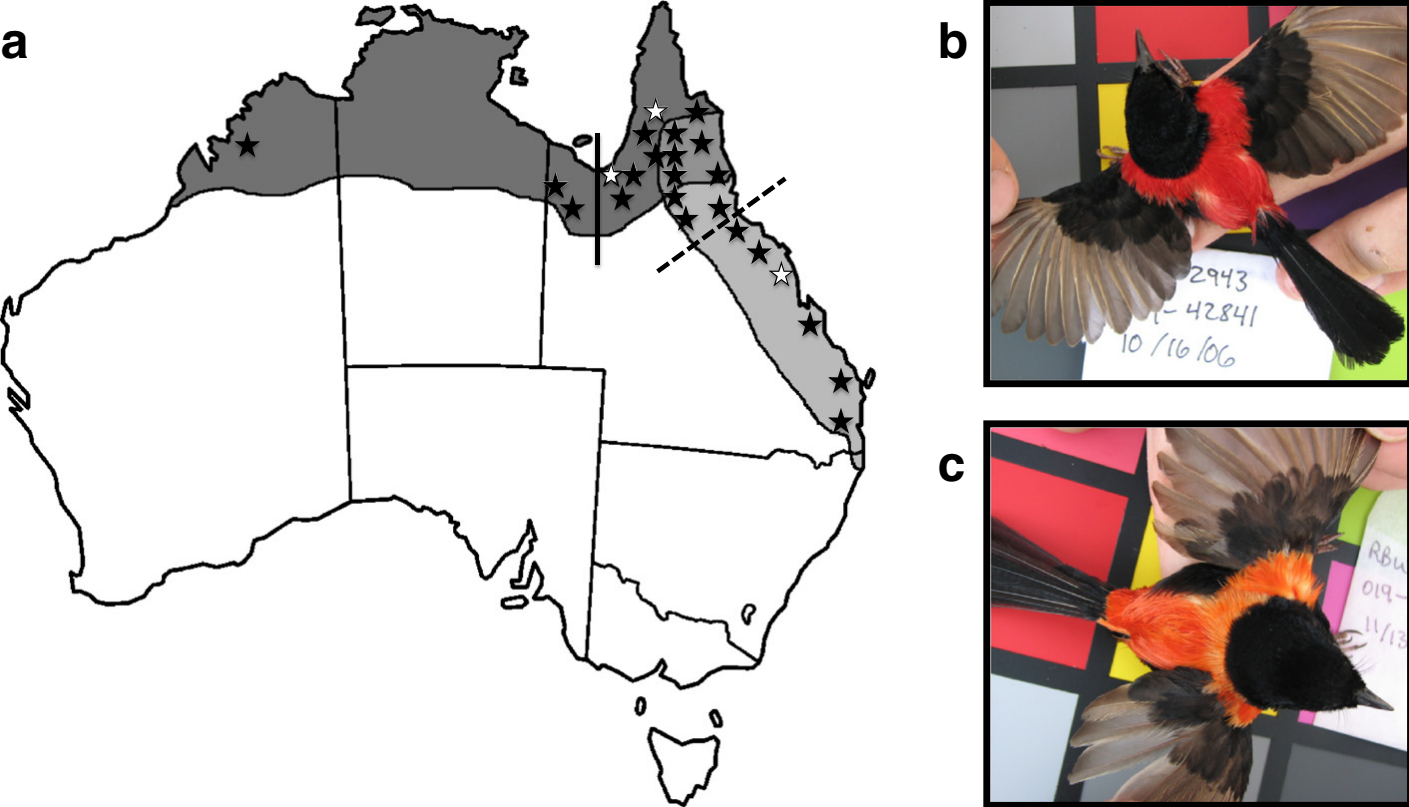
**The species range of the red-backed fairy-wren.** The species occurs across northern Australia, in the Cape York Peninsula, and along much of the east coast (**a**). The range of the crimson-backed, shorter-tailed *M. m. cruentatus* subspecies (**b**) is shaded dark grey and the range of the orange-backed, longer-tailed *M. m. melanocephalus* subspecies (**c**) is shaded light grey. Field observations have led to the subjective delineation of a morphological contact zone in the northeast. The solid line represents the Carpentarian Barrier, a biogeographic barrier across which the subspecies are genetically differentiated. The dashed line represents the eastern contact zone as we have defined it based on reflectance spectrometry of feather samples. Stars indicate sampling localities (N = 24) and white stars indicate three locations not included in the plumage color and male tail length dataset.

We examined these issues in the red-backed fairy-wren (*Malurus melanocephalus*), a small insectivorous passerine bird endemic to Australia. Currently there are two recognized subspecies that differ primarily in male plumage color and tail length [12]: the crimson-backed, shorter-tailed *M. m. cruentatus* subspecies occurs in northern Australia, and the orange-backed, longer-tailed *M. m. melanocephalus* subspecies occurs in eastern Australia (Figure 1). These subspecies are thought to also differ somewhat in morphometric traits, for example, *M. m. cruentatus* has been described as weighing less than *M. m. melanocephalus*[12]. Both subspecies are found in qualitatively similar open tropical savannah habitats across their entire range. There is a relatively large morphological contact zone between these subspecies in northern Queensland that is defined subjectively by the presence of males with intermediate values for tail length and plumage color [12]. Previous work has shown that male plumage color is a carotenoid-based [13] intersexual signal used by females during mate choice [14,15], and male tail length appears to be an intrasexual signal used primarily during male competitive interactions [16]. The two subspecies are moderately genetically differentiated across the Carpentarian Barrier, a well-known biogeographic barrier [17], suggesting divergence during the Pleistocene [18], likely followed by secondary contact.

Because the red-backed fairy-wren is at an early stage of speciation and exhibits variation in both sexual and non-sexual traits, it is an ideal species in which to explore the effects of the environment on divergence in sexual signals. In this study, we aimed to do this by analyzing variation in a suite of sexual and non-sexual traits across the species range and determining which traits are related to underlying environmental variation. Traits subject to ecological selection (such as morphometric traits that influence feeding behavior or locomotion) should correlate with variation in the physical environment. In contrast, traits subject to stochastic divergence in concert with sexual selection should not be correlated with environmental variation, but should instead exhibit geographic variation that is better explained by geographic distance, with greater distance signifying more time to accumulate stochastic variation (i.e., isolation by distance) [19]. By comparing the effects of environmental variables and geographic distance on sexual signals and morphometric traits, we aimed to distinguish between two alternative hypotheses: (1) if sexual signals have evolved via ecological selection, variation in these traits should be correlated with environmental variables, as is predicted for non-sexual morphometric traits; (2) in contrast, if sexual signals have evolved via sexual selection coupled with genetic drift, then this variation should be better explained by geographic distance, independent of environment. In addition, to further explore the geographic patterns of trait divergence between subspecies, we examined the influence of the Carpentarian Barrier and the plumage contact zone that exists further east (Figure 1) on trait dissimilarity (see methods for quantification of the eastern contact zone). Traits that have diverged between the two genetic lineages should show a strong signal of differentiation across the Carpentarian Barrier, as it is the hypothesized location of secondary contact and the genetic boundary between the subspecies [18].

## Results

### Morphometric variation

For all traits, we ran a model with environmental variables, geographic distance, and potential barriers as predictor variables (the full model); a model with only environmental variables as predictor variables; and a model with only geographic distance as a predictor variable. These were compared to models with random environmental variables to assess the significance of the results. For all morphometric traits, the full model explained much more variation in the data (range: 11.7-56.5%, mean = 32.1%, SD = 15.4, Table 1) than did the associated random model, (t-test comparing mean percent variation explained by full models and random models: *t* = 5.08, df = 7.59, p = 0.001). In all cases, the distance-only model was similar to the random model, with a low percent variation explained (range 0.9-11.5%, mean = 3.45%, SD = 3.7). For all but two morphometric traits, neither geographic distance nor either potential barrier was retained as an important predictor in the full model, and the environment-only and full models were thus identical. The full model for wing length did include geographic distance and the Carpentarian Barrier as important predictors, but they contributed relatively little to the fit of the model, improving the overall fit by only 0.7%. Similarly, the full model for bill width included the eastern contact zone as an important predictor, but it improved the overall fit by only 0.5%.

**Table 1:** Results of each generalized dissimilarity model for all phenotypic traits

	**Full model**		**Environment-only**		**Distance-only**	**Random variables**
**Trait**	**% var. explained**	**Predictor variables**	**% var. explained**	**Predictor variables**	**% var. explained**	**% var. explained**
Wing	31	(5) 2,6,7,10,16,17,18,20	30.3	(5) 3,6,9,13,16,17,18	0.9	4.1
Tarsus	46.5	(17,14,18,15) 4,6,7,10,11,16	46.5	(17,14,18,15) 4,6,7,10,11,16	2.1	2
Tail	31.4	(3) 2,5,6,7,8,9,13,16,18	31.4	(3) 2,5,6,7,8,9,13,16,18	11.5	3.9
Weight	56.5	(7,1) 9,11,14,15,16,17	56.5	(7,1) 9,11,14,15,16,17	0.9	9.9
Bill + head	38.9	(16,18) 2,3,5,7,10,11,14,15,17	38.9	(16,18) 2,3,5,7,10,11,14,15,17	6.4	0.7
Culmen	11.7	(7,11) 3,10,15,17	11.7	(7,11) 3,10,15,17	1	7.2
Bill depth	28.6	(7) 1,4,11,12,13,14,15,17	28.6	(7) 1,4,11,12,13,14,15,17	2.4	1
Bill width	13.4	(2,15,17,11) 3,7,18,21	12.9	(2,15,17) 3,7,11	2.4	3
Male tail	52.6	(5,10,3) 7,8,9,15,19	52.1	(5,10,3) 1,7,8,9,15,16	13.5	4.1
Plumage hue	78.9	(19) 2,3,6,7,9,11,15,21	62.6	(7) 1,2,4,9,11,12,15,16,17,18	47.6	10.4

For each trait, four separate models were run. Reported for each model are the percent of variation in the data explained, and the predictor variables that were retained in the model. Full models include isolating barriers if they were retained as important predictors. In parentheses are the most important predictor variables (with the highest response curve or response curve heights ≥ 50% of the highest), listed in order of relative importance. The remaining predictor variables are listed in numerical order. Variable numbers are taken from Table 2.

**Figure 2:**
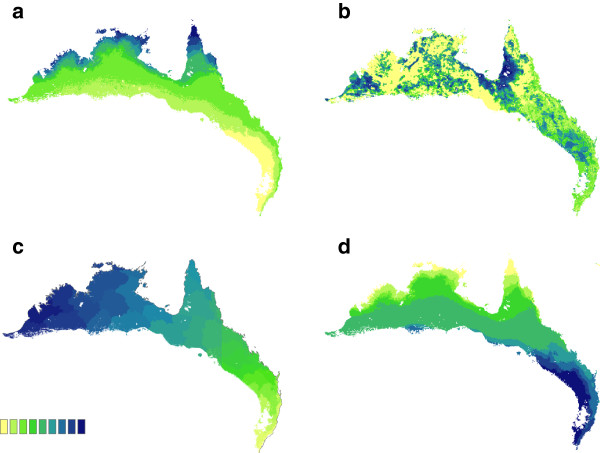
**Predicted spatial patterns of phenotypic variation.** Shown are model results for (**a**) wing length, (**b**) bill plus head length, (**c**) plumage hue, and (**d**) male tail length, as determined by the full generalized dissimilarity model for each trait. Maps for the remaining morphometric traits are not shown. Differences in color are proportional to differences in the trait value across the landscape (see color bar for scale).

**Figure 3:**
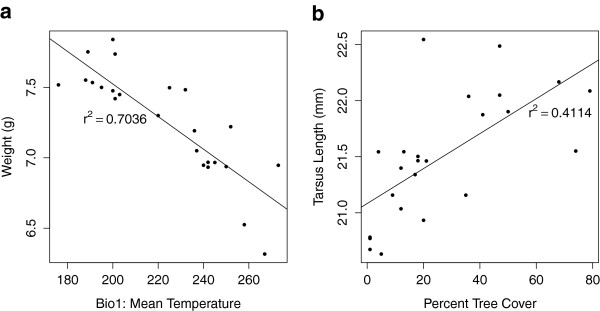
**Relationships between environmental variables and two morphometric traits.** Linear regressions of (**a**) weight (g) on Bio1: mean temperature, and (**b**) tarsus length (mm) on percent tree cover. Both linear regressions are significant at p < 0.001.

For each morphometric trait, different combinations of environmental variables were retained as important predictors, resulting in markedly different patterns of spatial variation (see Figures 2a and b for examples of predicted variation in morphometric traits). Although many morphometric traits were influenced by a combination of environmental variables considered important based on the height of their response curves (numbers in parentheses in Table 1), there were two models where a single environmental variable exhibited a response curve dramatically higher than any other. In the models of weight and tarsus length, this single environmental variable appeared to drive the overall pattern in each case: there was a strong negative relationship between weight and Bio1: mean temperature (linear regression, r^2^ = 0.70, p < 0.001, Figure 3a), and a strong positive relationship between tarsus length and percent tree cover (linear regression, r^2^ = 0.41, p < 0.001, Figure 3b). Finally, environmental variation explained significantly less variation in bill morphometric traits (culmen, width, and depth) than in other body morphometric traits (t-test comparing mean percent variation explained by GDM models of bill and non-bill morphometrics: *t* = 2.57, df = 5, p = 0.001).

### Plumage hue variation

**Figure 4:**
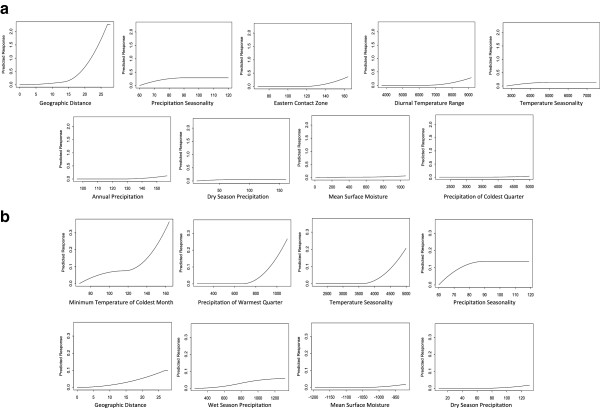
**Important predictor variables describing variation in sexual signals.** Response curves of input variables retained as significant predictors of variation in the full model of (**a**) plumage hue and (**b**) male tail length. Maximum height is indicative of the relative importance of each input variable, and the slope indicates the predicted rate of change in the response variable as a function of the predictor variable. Response curves for models of morphometric traits are not shown.

All models for plumage hue performed substantially better than the random model (Table 1). The distance-only model explained a substantial proportion of the variation in plumage hue (47.6%), and this variation explained was much greater than the variation explained by distance for any of the morphometric traits (mean = 3.45 ± 3.7%). The environment-only model also explained a substantial amount of variation (62.6%), but this pattern appeared to be driven by a spatial correlation between geographic distance and environmental dissimilarity. A linear regression of the most important predictor in the environment-only model (Bio15: precipitation seasonality) on geographic distance supports this idea (r^2^ = 0.24, p < 0.001, Additional file 1: Figure S3). Overall, the best fitting model for plumage hue also included the eastern contact zone as a predictor variable, and it improved the fit of the full model from 69.3% to 78.9%. In addition to the improvement of the fit by adding the eastern contact zone, the independent effect of geographic distance was the most important predictor variable in the full model (Figures 2c and 4a). In contrast, the Carpentarian Barrier – the boundary between genetic lineages – was not selected as an important predictor of plumage hue in any model.

### Male tail length variation

All models for male tail length performed better than the random model (Table 1). The distance-only model explained more variation in male tail length (13.5%) than in any other morphometric trait, although not as much as in plumage hue (above). The environment-only model explained 52.1% of the variation. The full model explained a similar amount of variation (52.6%), and although distance contributed to the fit of the model, it was not a very important predictor, and only increased the percent variation explained by only 0.5% (Figures 2d and 4b). Neither the Carpentarian Barrier nor the eastern contact zone was retained as an important predictor in the full model.

## Discussion

### Morphometric variation

As predicted, most morphometric traits were strongly correlated with environmental variables, suggesting a role for ecological selection in shaping their variation. The independent effect of geographic distance on variation in morphometric traits was only detected for wing length, and in this case the effect was minimal. Furthermore, the boundary between genetic lineages (the Carpentarian Barrier) was only selected as an important variable explaining variation in wing length, and again the effect was minimal. These results suggest that morphometric traits are not clearly divergent between the subspecies, but rather that geographic variation in these traits likely arises via local selective pressures from the physical environment, regardless of genetic background. This interpretation is further supported by close examination of the predicted spatial patterns of phenotypic variation. These traits are predicted to vary on a fine geographic scale in accordance with environmental dissimilarity across the landscape, and the resulting spatial patterns are quite different from what would be expected if one subspecies exhibited clear morphometric differentiation from the other. For example, variation in wing length follows a latitudinal gradient such that birds at similar latitudes appear to have similar wing lengths regardless of whether they are east or west of the genetic boundary between subspecies (Figure 2a).

GDM results describe how dissimilarity in phenotypic traits is associated with environmental dissimilarity but they do not provide information about the directionality of these relationships. However, examining the linear relationships between certain morphometric traits and important environmental variables revealed two interesting patterns. First, there was a strong negative relationship between weight and mean temperature. This result suggests that red-backed fairy-wrens may conform to Bergmann’s rule, which posits that organisms will evolve to be smaller in hotter climates to enhance thermoregulation via a more favorable surface area to volume ratio [20,21]. Second, there was a strong positive relationship between tarsus length and percent tree cover, and longer tarsi may be an adaptation enabling the red-backed fairy-wren to better maneuver and forage in trees sensu [22]. We stress that these relationships, although intriguing, are subject to multiple interpretations, and it is difficult to infer evolutionary mechanisms from correlations at such a broad scale. For example, in the case of tarsus length, greater tree cover may influence other aspects of the microhabitat (e.g., understory composition) not included in this study that may affect the evolution of tarsus length more directly.

Interestingly, the association between environment and bill morphology was much weaker than that for other (non-bill) morphometric traits, suggesting a relative lack of fit between bill morphology and environment. This pattern may be explained by the foraging ecology of the red-backed fairy-wren, as this species is a generalist insectivore capable of gleaning prey from leaves, foraging on the ground, and extracting prey from spider webs among other strategies [12, D. Baldassarre, personal observation]. Ecological selection on bill morphology may be relatively weak for a generalist of this sort, particularly if a single bill morphology is sufficient to exploit a diversity of prey. This interpretation is consistent with a recent analysis indicating a lack of ecological speciation among insectivorous warblers compared to granivorous finches [23].

### Plumage hue variation

In stark contrast to the patterns observed in morphometric traits, we found several lines of evidence supporting a diminished role of environment and a greater role of isolation by distance in explaining variation in plumage hue. First, the distance-only model for plumage hue explained much more variation in the data than did the distance-only models for morphometric traits. Second, when examining the full model for plumage hue, the independent effect of distance was evident, as it was by far the most important predictor of variation in plumage hue (Figure 4a). This pattern was maintained when the western-most sampling site was removed from the model (results not shown), indicating that the effect of geographic distance was not an artifact of a disproportionately distant sampling site. Finally, the predicted pattern of spatial variation in plumage hue (Figure 2c) was different from that of any morphometric trait: it did not vary in accordance with any of the environmental variables across the species range, but rather exhibited a clear change from one end of the species range to the other, with the greatest turnover occurring across the eastern contact zone. Taken together, these results indicate that isolation by distance is the most likely explanation for variation in plumage hue. Such a pattern is predicted by the hypothesis of divergent sexual selection via a Fisherian mechanism, coupled with genetic drift in signals and responses to those signals during the period when the two subspecies were geographically isolated [8]. Furthermore, isolation by distance is a particularly plausible mechanism of divergence in the red-backed fairy-wren, as they have extremely limited natal dispersal [24].

The finding that environmental variables have relatively little effect on geographic variation in plumage hue has several implications for the evolution and function of this sexual signal. First, it suggests that the observed divergence in the carotenoid-based plumage color that characterizes the subspecies is likely not a result of differential availability of carotenoids in the environment. If this were the case, we would expect to observe a pattern where red and orange birds occur in environmentally distinct habitats where there might be a different amount or composition of insect prey (these exclusively insectivorous birds likely acquire carotenoids from insect prey [12]). Although we have no data on insect prey diversity or abundances, and do not know which specific environmental variables influence carotenoid abundance, similar environmental data sources and modeling techniques have accurately characterized the distribution of at least two Australian insect species [25]. Moreover, although availability of carotenoids in the environment can affect plumage color in some species of birds [6], in many species, variation in carotenoid-based plumage color is not thought to be due to differential carotenoid intake [26,27], and a recent review concluded that carotenoid-based signals are no more likely to be environmentally influenced than melanin-based signals [28]. Similarly, availability of carotenoids does not appear to be responsible for variation in sexual signals in at least some other taxa [29]. Geographic variation in a carotenoid-based plumage signal might be affected by other mechanisms such as a difference in the timing of molt or usage of food resources between subspecies. However, there is no evidence of any such differences, and importantly, such mechanisms would also be imposed by variation in the physical environment, and the resulting association between environment and plumage color would have likely been detected by our methods, as was the case for morphometric traits. Thus, currently the most likely explanation is that geographic variation in the plumage signal is conferred by genetically based differences in the physiological ability to extract (from food), absorb, modify, transport, and deposit ingested carotenoids [30,31], though other explanations are possible. The most direct way to test this idea would be to directly modify the carotenoid content in the diet of captive birds from different geographic regions [6].

Second, these results also suggest that plumage color has not evolved through a sensory drive mechanism whereby a certain signal transmits more effectively in a particular physical environment sensu [32]. However, it is possible that the environmental variables included here may not be relevant to color transmission. Analysis on a finer spatial scale with different environmental characteristics (e.g., ambient light) might reveal differences in microhabitat not detected in this study.

Isolation by distance rather than ecological selection seems to be influential in shaping variation in plumage hue, but the best-fitting model also included the eastern contact zone as a predictor variable, indicating that there is a high degree of dissimilarity in plumage hue across this region. This area, however, is located approximately 300 km east of the boundary between genetic lineages, the Carpentarian Barrier, across which we detected no significant difference in plumage hue. We suggest that the most likely explanation for this mismatch between the genetic boundary and the phenotypic contact zone is the asymmetrical introgression of red plumage from the western *M. m. cruentatus* subspecies into the genetic background of the eastern *M. m. melanocephalus* subspecies. Theory supports the idea that hybridization can facilitate the introgression of an advantageous trait into another population [33-35]. In this case, introgression of red plumage may be driven by sexual selection if red-backed males have a sexually selected advantage via male competition [36] or female choice [37]. Finer-scaled spatial analyses of the possible lack of coincidence between genetic and phenotypic clines as well as field experiments will be necessary to test this idea.

### Male tail length variation

Male tail length is presumed to be an intrasexual signal used in male competition [16], and we predicted that it would exhibit a pattern of variation similar to plumage hue. Instead, the pattern of variation in male tail length was more similar to those of morphometric traits, where environmental variables were retained as important predictors of variation, but geographic distance was not. In fact, the predicted pattern of spatial variation in male tail length was very similar to that of wing length (both are predicted to follow a latitudinal gradient, compare Figures 2a and 2d). Geographic distance explained more variation in male tail length than in the morphometric traits, but this effect was not as strong as in the models of plumage hue. This pattern suggests that evolution of male tail length may be constrained by ecological selection if tail length impacts survival in some way. Red-backed fairy-wrens are not strong fliers and maneuver through their habitat primarily by hopping and making short flights [12, D. Baldassarre, personal observation], so the tail may play an important role in stability and balance during locomotion. This constraint may explain why there is not a clear divergent pattern of isolation by distance as is seen in plumage hue, despite a trend for *M. m. cruentatus* males to have shorter tails than *M. m. melanocephalus* males [12].

## Conclusions

In summary, comparing the influence of environment on sexual and non-sexual traits suggests that different evolutionary forces have shaped geographic variation in these traits. The red-backed fairy-wren exhibits variation in a number of morphometric traits such as weight and wing length that is well explained by environmental variation but does not show a clear pattern of divergence between the subspecies. These results suggest that ecological selection has acted on these traits to create geographic variation that is independent of subspecies identity, such that individuals exhibit morphometric traits that are adapted to the local environment. In contrast, variation in plumage hue, the most salient difference between the subspecies, is not well explained by environment, but rather shows a strong pattern of isolation by distance across the species range, with a particularly high rate of plumage hue change across the eastern contact zone. This pattern suggests that geographic variation in hue likely has evolved via a Fisherian mechanism, beginning with a period of stochastic divergence [8]. Upon secondary contact, red plumage appears to have introgressed across the genetic boundary between the subspecies, possibly driven by sexual selection. The evolutionary forces acting on male tail length appear to be more complex, as variation in this putative signal seems to be constrained to some extent by the environment, and male tail length has likely not evolved purely via stochastic or sexually selected processes. This study highlights the importance of considering sexual selection in combination with stochastic processes when examining the evolution of sexual signals, including those predicted to be under strong environmental influence. In addition, there is evidence that two putative sexual signals have evolved via different evolutionary pathways, with variable selective pressures from the physical and social environments. Further experimental studies are needed to test the information content and function of male plumage color and tail length in multiple populations.

## Methods

### Field methods

Between 2004 and 2011, we captured 480 adult red-backed fairy-wrens at 24 sites throughout their range (Figure 1a). We captured birds using mist nets, weighed them to the nearest tenth of a gram using a spring scale, and measured the following morphometric traits to the nearest tenth of a millimeter with digital calipers: wing length, tarsus length, tail length, exposed culmen length (the length from the tip of the bill to the middle of the nares), bill width and depth (measured at the middle of the nares), and the total length of the bill plus head (distance from the back of the head to the tip of the bill). Feather samples from the center of the back (which are colored red-orange) were taken from all adult males in breeding plumage (N = 241) for color quantification using reflectance spectrometry. Our plumage color and male tail length data set was restricted to 21 sites, because no males in breeding plumage were captured at three sites (Figure 1a). In our analyses of non-sexual morphometric traits, “tail” refers to tail length of males and females combined, and includes birds from all 24 sites.

### Reflectance spectrometry and plumage color analysis

We used reflectance spectrometry to objectively measure plumage color variation. From each individual male’s feather sample, we mounted six feathers in an overlapping pattern on a square of black construction paper (Strathmore Artagain® Coal Black). To measure reflectance of the feather sample, we used an Ocean Optics USB2000 UV–VIS spectrometer with an R200-7 UV–VIS probe, and a PX2 pulsed xenon light source. The probe tip was mounted in a metal block to exclude ambient light, and the probe illuminated a measurement area of 3 mm^2^ on the feather sample. Three reflectance curves were generated for each sample, and the probe was re-calibrated against a white standard (Ocean Optics WS-1) after each individual. We averaged the three reflectance curves to produce one curve per individual, and the reflectance in the avian visible spectrum (300 nm-700 nm) was analyzed.

To obtain color metrics accounting for the spectral sensitivity of the avian visual system, we used the program TetraColorSpace [38], which analyzes reflectance curves using the spectral sensitivity of each of the cones in the avian retina, and plots each color as a point in a tetrahedral color space. The red-backed fairy-wren has a violet-sensitive visual system [39] so we analyzed feather samples using the average avian violet-sensitive spectral sensitivity curve [40]. For the present analysis, we used the color metric θ (theta), which describes plumage hue. Theta is defined as the angular displacement of the color vector from the positive X-axis of the tetrahedral color space, which runs between the green and red vertices [38]. We chose this metric because it best captures the geographic variation in plumage color (i.e., there are no consistent differences in plumage brightness or chroma across the species range; unpublished data). Theta can be interpreted as the direction of the color vector, and quantifies, in this case, orange vs. red plumage [38].

### Delineation of habitat range using species distribution modeling

To delineate a suitable habitat range for the red-backed fairy-wren, we used the “maximum entropy” species distribution modeling approach employed in the program Maxent 3.0 [41]. Maxent 3.0 uses presence only data plus environmental variables at sites where the species has been recorded. We used 6,865 localities of known species occurrences, from an initial total of 18,595 records from BirdLife Australia (http://www.birdata.com.au/maps.vm); the initial dataset was reduced by only including a single known locality per 30 × 30 arcsec grid cell. Maxent 3.0 returns logistic probabilities for each grid cell, with increasing values indicating higher probabilities of species occurrence. These values were then converted to a presence/absence map using the threshold “balance training omission, predicted area and threshold value” [41]. We used the following settings in Maxent 3.0: 1,000 background points, auto features, regularization multiplier = 1.0, maximum iterations = 500, convergence threshold = 0.00005. Because we were mainly interested in a relatively broad delineation of the distribution of the red-backed fairy-wren, rather than identifying the limiting conditions of their distributions or the distributions of sub-species, we did not perform further tests of the Maxent model. This will be the subject of a future study. The resulting model had an AUC (area under the receiving operator curve) [41] value of 0.77, and describes an area of suitable habitat consisting mainly of open tropical savannah (Additional file 1: Figure S1). The model is highly consistent with the previously estimated species range of the red-backed fairy-wren [12].

### Predictor variables included in models

In an attempt to capture biologically meaningful variation in the physical environment across the range of the red-backed fairy-wren, we included data from several ground-based and remotely sensed sources (Table 2). Eleven bioclimatic variables from the WorldClim database (http://www.worldclim.org) were included that represent annual means, seasonality, and seasonal extremes in temperature and rainfall [42]. These data were collected from weather stations and represent climatic variation between 1950–2000 that has been shown to accurately characterize the species ranges of Australian taxa [43]. In addition to these bioclimatic variables, we also included a suite of remotely sensed environmental variables in our analyses. The QuickScat product (http://www.scp.byu.edu) uses active radar scatterometers to measure surface moisture and is insensitive to cloud cover. High-resolution elevation data was gathered from the Shuttle Radar Topography Mission (SRTM). Finally, from the moderate resolution imaging spectroradiometer aboard the MODIS satellites (http://modis.gsfc.nasa.gov), we included data on percent tree cover and photosynthetic activity as measured by the normalized difference vegetation index (NDVI). To enable simultaneous comparison of the effects of all environmental variables, those with resolutions higher (e.g., SRTM, 30 m) or lower (e.g., QuickScat, 2.25 km) than 1 km were re-aggregated to a 1 km grid cell resolution. Geographic distance was computed as the shortest straight-line distance between two sampling sites.

**Table 2:** The 21 predictor variables included in this study

**Variable number**	**Data source**	**Variable name**	**Variable interpretation**
1		Bio1	Mean temperature
2		Bio2	Diurnal temperature range
3		Bio4	Temperature seasonality
4		Bio5	Maximum temperature of warmest month
5		Bio6	Minimum temperature of coldest month
6	WorldClim database	Bio12	Annual precipitation
7		Bio15	Precipitation seasonality
8		Bio16	Wet season precipitation
9		Bio17	Dry season precipitation
10		Bio18	Precipitation of warmest quarter
11		Bio19	Precipitation of coldest quarter
12		NDVImax	Maximum vegetation production
13	MODIS satellite spectroradiometer	NDVImean	Mean vegetation production
14		NDVIsd	Vegetation seasonality
15	QuickScat active radar scatterometer	Qscatmean	Mean surface moisture
16		Qscatsd	Surface moisture seasonality
17	Shuttle Radar Topography Mission	Elevation	
18	MODIS satellite spectroradiometer	Percent tree cover	
19		Geographic distance	
20	GPS	Carpentarian barrier	
21		Eastern contact zone	

We considered the influence of 18 environmental variables, geographic distance, the Carpentarian Barrier, and the eastern contact zone on phenotypic variation. Variable numbers are referred to in Table 1.

To examine the importance of the Carpentarian Barrier and the eastern contact zone in explaining trait divergence, we introduced a binary GIS layer for each region as a predictor variable in the full model, with values equal to 0 west of the region and 1 east of the region. Thus, comparisons between sites across the region receive a value of 1 for this predictor variable, while comparisons between sites on the same side of the region receive a value of 0. Preliminary analysis of variation in plumage hue across the red-backed fairy-wren range suggested that the eastern contact zone between plumage types is in fact located further down the east coast than is suggested by the previously published species range (Additional file 1: Figure S2). Thus, we used the area defined by the approximate center of the plumage cline as the eastern contact zone in our models. Rigorous cline-based analyses of spatial variation in plumage hue will be the subject of a future study.

### Generalized dissimilarity modeling of phenotypic variation

To analyze the effect of environmental variation on phenotypic variation across the red-backed fairy-wren range, we used generalized dissimilarity modeling (hereafter GDM) [44]. GDM is a matrix regression technique that predicts biotic dissimilarity across the landscape based on the matrix correlation between biotic dissimilarity and environmental dissimilarity plus geographic distance between sites where the species has been sampled. GDM can fit non-linear relationships between environmental variables and phenotypic traits using I-spline basis functions, and also can consider the effect of geographic distance independent of environmental variables. This is an important feature of GDM that controls for the often-observed correlation between geographic distance and environmental dissimilarity by analyzing the independent effect of distance. GDM is a two-step process. First, dissimilarities in predictor variables (i.e., environmental variables, geographic distance, and potential isolating barriers) between all pairwise combinations of sampling sites are fit to dissimilarities in response variables (i.e., phenotypic traits). For all phenotypic traits, we computed dissimilarity between sites as the Euclidian distance between the average trait values at each site divided by the sum of the standard deviation at each site, to control for within-site variability. The relative contribution of each predictor variable is tested using a permutation with the following structure: The predictor variables are introduced to the model in random order and the variation in the response variable explained by the inclusion of that variable is compared to that without the variable (ΔD). Next, the predictor variable is added again in a large number of random permutations of the order of sampling locations, resulting in a random distribution of ΔD [44]. ΔD resulting from the inclusion of the properly seeded response variable is compared to that distribution, and the variable is retained if ΔD is significantly higher than the random distribution. Second, using these results, a spatial pattern of response variables is predicted across the entire range as selected by the species distribution model described above. This pattern is color-coded, with differences in color proportional to differences in the response variable. The relative importance of each predictor variable can be assessed by response curves, where the maximum height is indicative of the relative importance, and the slope indicates the predicted rate of change in the response variable as a function of the predictor variable. We considered predictors retained in the model particularly important if they exhibited a response curve with a height ≥ 50% of the predictor with the highest overall response [45].

To consider the possibility of a correlation between geographic distance and environmental dissimilarity, we ran three separate models for each response variable with the following predictor variables: a full model with environmental variables, geographic distance, and potential isolating barriers; a model with only environmental variables; and a model with only geographic distance. To examine the fit of the models compared to the null hypothesis of no influence of any predictor variable, we also ran a model with random values of environmental variables in each grid cell and the variation explained by the full model was compared to the random model [45]. Computational limitations prevented the creation of a distribution of random models for each trait, but the difference in explanatory power between the random and full models was quite large (typically an order of magnitude, see results), thus we felt confident evaluating the strength of our models compared to one iteration of the random model.

## Competing interests

No authors have any competing interests, financial or otherwise, that should prevent the publication of this work.

## Authors’ contributions

DTB collected samples in the field, analyzed feather samples, compiled datasets, and drafted the manuscript. HAT ran the Maxent 3.0 and GDM models. JK and MSW participated in the design of the study and collected samples in the field. All authors read and approved the final manuscript.

## Supplementary Material

Additional file 1 Supplemental information. Click here for file
