# Supplementary material for: The role of ecological variation in driving divergence of sexual and non-sexual traits in the red-backed fairy-wren (Malurus melanocephalus)
Source: BMC Evol Biol. 2013 Mar 28;13:75. doi: 10.1186/1471-2148-13-75 (PMC3639809; doi:10.1186/1471-2148-13-75)
Supplement: Additional file 1 — Supplemental information. [file 1471-2148-13-75-S1.docx]

**Supplemental Information**

**Figure S1: Distribution of the red-backed fairy-wren as modeled by Maxent 3.0.**

Warmer colors indicate more suitable environmental conditions; cooler colors indicate less suitable conditions. See text for details on model parameters.

**Figure S2: Plumage hue across the red-backed fairy-wren species range.**

The western-most sampling location (Mornington) was assigned distance 0, and distance 3052 km represents the sampling location in the far southeast of the species range. Distances were calculated as the shortest straight-line distance between a given location and Mornington except in cases where this path would cross into unsuitable habitat as determined by the species distribution model (see Figure S1). In these cases, the distance was calculated as the straight-line distance from Mornington to a “pivot point” on inner edge of the range, plus the straight-line distance from the “pivot point” to the given location. Values for theta (hue) were converted to absolute values for visualization purposes, with larger values indicating redder hue. The result is a cline describing a transition from redder plumage in the west to more orange plumage in the southeast. The approximate center of the cline was computed as the distance along the cline that encompassed the median theta value (dashed line). This region was used as the eastern contact zone in GDM analyses. The solid line represents the genetic boundary between subspecies, the Carpentarian Barrier.

**Figure S3: The spatial correlation between geographic distance and environmental dissimilarity across the red-backed fairy-wren species range.**

Linear regression of pairwise difference in Bio15: precipitation seasonality on geographic distance among sites in the RBFW species range (p < 0.001).
